# The economic disease burden of measles in Japan and a benefit cost analysis of vaccination, a retrospective study

**DOI:** 10.1186/1472-6963-11-254

**Published:** 2011-10-07

**Authors:** Kenzo Takahashi, Yasushi Ohkusa, Jong-Young Kim

**Affiliations:** 1 Clinical Research Center Sanno Hospital, International University of Health and Welfare, 8-10-16 Akasaka, Minato-ku, Tokyo, 107-0052, Japan; 2 Department of International Health Cooperation, Japan(IMCJ), National Center for Global Health and Medicine, 1-21-1 Toyama, Shinjuku-ku, Tokyo,162-8655, Japan; 3 Infectious Disease Surveillance Center, National Institute of Infectious Diseases, 1-23-1 Toyama, Shinjuku-ku, Tokyo, 162-8640, Japan; 4 Department of Pediatrics, Chiba-Nishi General Hospital, 107-1 Kanegasaku, Matsudo-shi, Chiba, 270-2251, Japan

## Abstract

**Background:**

During 1999-2003, Japan experienced a series of measles epidemics, and in *Action Plans to Control Measles and the Future Problems*, it was proposed that infants be immunized soon after their one-year birthday.

In this study, we attempted to estimate the nationwide economic disease burden of measles based on clinical data and the economic effectiveness of this proposal using the benefit cost ratio.

**Methods:**

Our survey target was measles patients treated at Chiba-Nishi general hospital from January 1999 to September 2001. Two hundred ninety-one cases were extracted from the database. The survey team composed of 3 pediatricians and 1 physician from Chiba-Nishi general hospital examined patient files and obtained additional information by telephone interview.

We analyzed data based on a static model, which assumed that the number of measles patients would be zero after 100% coverage of single-antigen measles vaccine.

Costs were defined as the direct cost for measles treatment, vaccination and transportation and the indirect cost of workdays lost due to the nursing of patients, hospital visits for vaccination or nursing due to adverse reactions. Benefits were defined as savings on direct and indirect costs. Based on these definitions, we estimated the nationwide costs of treatment and vaccination.

**Results:**

Using our static model, the nationwide total cost for measles treatment was estimated to be US$ 404 million, while the vaccination cost was US$165 million. The benefit cost ratio of the base case was 2.48 and ranged from 2.21 to 4.97 with sensitivity analysis.

**Conclusions:**

Although the model has some limitations, we conclude that the policy of immunizing infants soon after their one-year birthday is economically effective.

## Background

Japan is one of the countries most affected by measles, a contagious disease with many complications. The measles vaccine was first introduced to Japan in 1966 and was adopted in the national regular immunization program from 1978 [1]. Before April 2006, when Japan adopted two-dose MR vaccine policy, Japan's *Preventive Vaccination Act *made provisions for single-antigen attenuated live vaccine to be given only once to children aged 12-90 months. Nationwide coverage remained no higher than 81%. Since 1994, the government of Japan has seemed very passive in controlling vaccine preventable diseases, as the vaccine policy was changed from being a compulsory immunization to being voluntary. As a consequence, measles vaccine coverage rates have been lower than other countries [2,3].

**Figure 1:**
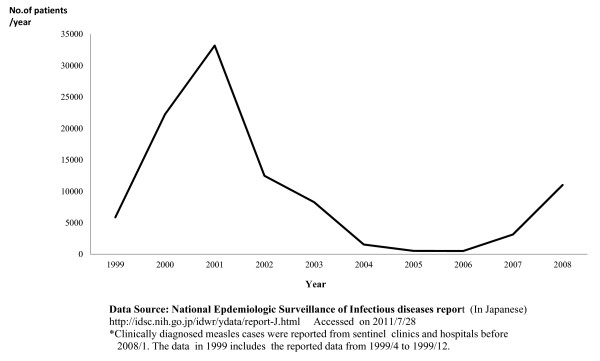
**Reported Number of Measles Patients**.

Between 1999 and 2003, Japan experienced a series of measles epidemics. During these epidemics, the number of reported cases ranged from 5,957 (1999) to 34,734 (2001) [4] (Figure 1). There were approximately 100,000 to 200,000 estimated cases during this time [3,5]. During 1999-2007, measles surveillance in Japan consisted of aggregate case reporting systems from pediatric and adult sentinel surveillance systems in which pediatric cases were reported from a representative reported sample of approximately 3,000 pediatric inpatient and outpatient facilities and adult cases were reported from a sample of approximately 450 inpatient hospitals. From these reports, the total number of measles cases was estimated. Measles sentinel reporting systems were replaced with nationwide case-based reporting system in January 2008 [4].

The measles epidemics of 1999-2003 were attributed to insufficient disease suppression due to low vaccination coverage, which ranged from 75 to 81% [6,7]. They had two characteristics: they were all small-medium in epidemic size [8], and the main victims were unvaccinated 1-year-old children. In the nationwide survey, the estimation of 2002 measles vaccination coverage in Japan revealed that Japan's measles vaccine coverage at ages 18, 24, and 36 months were 61.7 ± 1.6%, 79.6 ± 1.3%, and 86.9 ± 1.1%, respectively [9]. The coverage of 18-month-olds was revealed to be rather low for protection from measles transmission, presumably making the group susceptible to measles infection. The age distribution of measles patients supported this presumption because one of the peak ages for measles patients was one year. The National Institute of Infectious Diseases, Japan (NIID) reflected on these characteristics in a publication entitled, *Action Plans to Control Measles and the Future Problems *[6] and proposed that infants be vaccinated as soon as possible after their one-year birthday to reduce the age group's susceptibility to measles infection. Even though the two-dose regimen is favored around the world, the NIID recommendation was considered the best possible rapid option under laws governing immunization regulations. It was adopted by the Ministry of Health, Labour and Welfare, Japan with the combined support of the Japan Pediatric Association, the Japan Child Health Association, and the Japanese Association of Pediatrics [10].

In Japan, the regular immunization service, which includes the measles vaccine, is given by medical doctors in hospitals and clinics. Under such programs, local governments request support from local medical associations, which are typically comprised of physicians in private clinics that are governed by the Japan Medical Association. Local medical associations delegate responsibilities to eligible doctors and parent(s) take their children to clinics for immunization. Participating doctors are paid for their participation. The amount is variable, as it is determined by the individual local governments. Hospitals and clinics in charge of immunization must procure vaccine, syringes and needles at their own expense. Usually, vaccine delivery costs are included in vaccine price. This system is in place throughout the country.

Japan's medical service system differs to that of the United States and many European countries in that patients are generally seen without appointment [11]. Because the referral system between GPs and hospitals is not well established, many patients go directly to hospitals without appointment, and measles patients are often first attended by doctors in hospitals. There are typically two options for hospital admission: patients may be admitted directly after consultation with a physician in the outpatient ward of a hospital; or they may be referred by a GP. Admission fees are standardized under the national health insurance program but the cost of private beds varies - as it is determined by the individual hospitals.

In the present study, we tried to estimate the nationwide economic disease burden of measles based on the clinical data of local measles epidemics in Matsudo City, Chiba Prefecture, Japan between 1999 and 2001. At the same time, we attempted to evaluate the economic effectiveness of the proposal that infants be immunized soon after their first birthday by benefit cost ratio (BCR).

Even though Japan is considered a measles endemic country, health policy research about this topic is still missing and to date, an economic evaluation on the cost of the disease has not been performed. This is the first policy evaluation of Japan's measles vaccination policy based on an economic viewpoint.

## Methods

We performed a retrospective study of Chiba-Nishi General Hospital patients identified by a file survey. After complementing some of the data through telephone interviews, direct costs and indirect costs were analyzed following the framework of analysis published by Ohkusa *et al *[12] and Sugawara *et al *[13]. Finally, nationwide costs and BCR were estimated based on a static mathematical model of measles transmission [14].

### Study design

This study is based on the assumption that the number of measles patients would be zero if all 1-year-old cohorts received the vaccine. We used a static model, as such we did not consider the adjustment period of gradual herd immunity increase and final measles epidemic control. Regarding the framework of the analysis, for the cost-benefit analysis of influenza vaccination we followed the methodology published by Ohkusa et.al [12] and for the cost-effectiveness analysis of routine immunization for varicella we followed the framework of Sugawara *et al *[13].

### Survey area

The data were sampled from patient records from Chiba-Nishi General Hospital from January 1999 to September 2001, where two of the authors worked as pediatricians. This private hospital is located at Matsudo City, which is adjacent to metropolitan Tokyo and has a population of about 470,000. Regarding the health facilities of Matsudo City, there are 13 pediatric clinics and 153 GP clinics, in which 59 physicians also see pediatric patients. Out of 13 general hospitals in Matsudo-city, 3 hospitals, including Chiba-Nishi General Hospital, have a pediatric outpatient service. Chiba-Nishi General hospital has 408 beds, a pediatric outpatient ward, an inpatient ward and an emergency unit. It receives an average of 900-1,000 patients per day, including 200-300 pediatric outpatients.

### Case definition of measles

Firstly, we referred the diagnostic criteria of Japan's sentinel surveillance. The diagnostic criteria of sentinel surveillance for measles included: the presence of a generalized rash; fever (≥ 38.5°C); and cough, coryza, or conjunctivitis; or laboratory confirmation. Laboratory confirmation of cases was performed by detection of measles-specific immunoglobulin M (IgM) antibodies [4].

In our study, selection criteria were: 1) cases diagnosed as measles by measles IgM testing; or 2) cases diagnosed as measles by Koplik spots. Koplic spots were included as diagnostic criteria because the presence of Koplik spots is most important in establishing the diagnosis of measles [15]. Furthermore, all of the clinical records of the cases were thoroughly examined by the survey team to determine whether the clinical course was consistent with measles and confirm the measles diagnosis.

### Data collection

Data was collected between October 1 and October 28, 2001. For the first stage of data collection, cases were extracted from the patient diagnosis electronic database, which was developed to assist in the claiming of national health insurance; it includes information on both confirmed and suspected cases. We examined the database from January 1999 to September 2001.

For extraction of data, we allocated two qualified medical clerks, who were briefly instructed and trained in the data extraction procedures to ensure data coherence. Two hundred and ninety-one cases were extracted from a total of 375,353 records. In the second stage, relevant patient files were examined by our survey team, which included one of the hospital's physicians and three of the hospital's eight pediatricians. Research target candidates were nominated if the records met the diagnostic criteria noted above in "Case definition of measles".

The data that were initially collected included patient name, age, gender, course of fever, clinical symptoms other than fever, contents of the examination and treatment, dates of hospital visit, contents of medical examination and treatment, fees for medical examination and treatment, patient/parental employment status, address and telephone number, and for inpatients, the date of admission and discharge, contents of treatment and examination, and the fee paid by national health insurance. In order to respect patient privacy, access to any identifying information that was necessary for the study was maintained for only as long as it was needed. Once relevant estimations could be made, sensitive information was deleted. Information on employment status was deleted after estimating indirect costs. Patient addresses were only used to calculate transportation fees and were not recorded as the calculation was performed immediately after case selection.

Telephone interviews were conducted to follow up with patients whose records of parental employment status were not clear or where prognosis was not identified due to discontinuation of treatment at the hospital. The three above-mentioned pediatricians, who were in charge of treating pediatric outpatients at Chiba-Nishi General Hospital, performed the interviews. Before beginning the telephone interview, the exact purpose of this survey was explained to the interviewees. Information was only recorded after obtaining verbal consent from the interviewee. In the interview, parent(s) were asked the date of the onset of rash, the duration of the rash, the course of fever and whether their child experienced any other complications. No other sensitive or identifying information was collected. Following the telephone interview, patient identity and telephone numbers were deleted.

### Framework of analysis

To estimate the costs and benefits, it is important to define who should assume the cost, and who should receive the benefits. For our research objective, we aimed to provide an estimation that was applicable to the whole of Japan.

We defined direct costs and indirect costs, which are hereafter defined in "Definition of costs". Nationwide direct and indirect costs based on these definitions were then estimated from our sample data.

### Definitions of costs

Costs were categorized into direct cost and indirect cost (Table 1). Direct costs are defined as: 1) fee for vaccination and transportation fees for hospital visit; 2) actual fees paid for medical diagnosis and treatment of measles and hospital visit. Indirect costs were defined as workdays lost due to the nursing of measles patients and workdays lost due to vaccination or nursing for mild side effects of vaccination; this also includes any productivity losses due to any measles-related deaths. While vaccination cost incurs yearly as a control cost of measles, measles treatment costs don't incur yearly because the incidence may be reduced to be zero based on our model.

**Table 1:** Definition of costs and benefits

Costs	Direct Costs	Vaccination Fee Transportation Fee
	
	Indirect Costs	Work days lost for immunization Work days lost due to nursing for adverse reactions
Benefits	Reduction of direct costs	Medical treatment fee(including admission fee) Transportation
	
	Reduction of indirect costs	Work days lost by patients and family members Lost income due to death and severe adverse reactions

These costs are estimated based on several assumptions that will be discussed later in this text. Benefits are defined as reductions of direct and/or indirect costs. Nationwide direct and indirect costs were estimated based on our sample data and the BCR was based on these estimations.

For precise data, currency conversions should reflect the monthly average of the exchange rate. However, for simplicity we opted to use the average exchange rate of the study period (US$ 1 = JP¥ 118.8).

### Direct costs

#### Medical treatment fees

Costs for medical consultation, prescribed medicines, laboratory examination and X-ray examinations were included as medical fees. Admission fees were included for inpatient cases. We followed the national standard for reimbursement of medical services as medical treatment fees.

Since there is no standardized treatment and diagnosis for measles or its complications, several tests and treatments were found to have been used in the sample cases. Thus, the diagnosis and treatment of each case were thoroughly examined to ensure that only those that were medically appropriate were selected for analysis. For the sake of simplicity, the cost of over-the-counter drugs was not included. Furthermore, we did not consider cost of vaccine delivery because this is included in the vaccine procurement cost, which is reimbursed by local governments.

#### Transportation fees for hospital visits

For transportation fees of patients or their attendants, we estimated the cost incurred by public transportation using the patient's address and the public transportation routes to the hospital. As previously noted, patient addresses were checked from patient files before data were extracted, in order to estimate these costs but not compromise privacy. Address information was not recorded.

### Indirect costs

The estimation of indirect costs included: 1) Estimation of wage functions; 2) assumption settings of indirect costs; and 3) estimation of workdays lost by patients or family members. The procedure for the estimation of wage function is described in detail in additional file 1 (see also Tables 2 and 3).

**Table 2:** Estimation of Wage Function

Explanatory variable	Male		Female		Part-time worker**			
	
	Estimated Value	Probability	Estimated Value	Probability	Estimated Value	Probability	Estimated Value	Probability
Age	0.106	0.000	0.0480	0.000	0.001	0.756	0.0003	0.687
Age^2^	-1.08 × 10^-3^	0.000	-5.55 × 10^-4^	0.000	-1.70 × 10^-5^	0.804		
Constant term	3.42	0.000	4.41	0.000	6.743	0.000	6.77	0.000

No. of Samples*	12		12		12		12	
F static	169		37.6		0.110		0.170	
Probability	= < 0.000		= < 0.000		0.896		0.687	
Coefficient of determination	0.976		0.893		0.024		0.016	
${\bar{R}}^{2}$	0.971		0.869		-0.193		-0.081	

Note that Table 2 is cited in the additional file 1(Technical Annex).

For regular employee, explained variable is log (prescribed monthly salary/1,000)

For part-time worker, explained variable is log (prescribed per-hour wage)

*The value 12 quotes the 12 age classification used in census of earnings.

** 2 estimated values of Part-time worker mean that part time worker's wage is not expressed by the function of age respectively

**Table 3:** Estimation of the indirect cost function of patient attendance

Explanatory variable	Estimated Value	Probability	Estimated Value	Probability	Estimated Value	Probability	Estimated Value	Probability
Patient' age	0.0223	0.0300	-0.0345	0.189			0.00380	0.934
Patient' age 2	1.00 × 10^-4^	0.669	4.03 × 10^-3^	0.0170	1.80 × 10^-3^	0.000	-5.00 × 10^-4^	0.905
Patient' age 3			-6.600 × 10^-5^	0.0190	-3.10 × 10^-5^	4.00 × 10^-2^	0.0011	0.529
Patient' age 4							-2.16 × 10^-6^	0.311
Constant term	8.65	0.000	8.73	0.000	8.67	0.000	8.69	0.000

No. of samples	99		99		99		99	
F statistic	48.8		36.5		53.3		27.2	
Probability	= < 0.000		= < 0.000		= < 0.000		= < 0.000	

Note that Table 3 is cited in the additional file 1(Technical Annex).

99 samples were selected based on data reliability of age and employment status.

One sample was omitted because parental age and employment status were unclear.

#### Estimated wage function

We calculated the following wage functions following the procedure summarized in additional file 1.

Male regular employee

ln {wage_(yen)_} = 3.418+0.106 × (*Age*)-0.00108 × (*Age*)^2^

Female regular employee

ln {wage_(yen)_} = 4.406+0.048 × (*Age*)-0.000555 × (*Age*)^2^

Part-time worker

Wage/hour _(yen) _= JP¥ 880 = US$ 7.4 (Exchange rate: US$ 1 = JP¥ 118.8)

### Base case setting

In benefit-cost analysis, as a reference standard, the base case should be composed using plausible parameters. In addition, we conducted a sensitivity analysis by changing certain parameters. The base case was set according to the economic situation in Japan in 1999-2003 [8].

The target population for immunization of 1,200,000 per year reflects the vital statistics of Japan [16]. The vaccination fee was set at JP¥ 5,000 (US$ 42.1)/person which were disbursed to each of the vaccinating physicians as compensation from the public health offices of local governments in Matsudo-city. To receive the vaccine, one of the parents should take off two workdays (the day of vaccination and the next day). For homemakers the absence includes the suspension of housework. For these 2 days, we assumed that an immunized child may have fever and that in such cases one parent must stay home to provide care. This is based on the fact that 20% of vaccine recipients suffer from mild fever lasting for approximately 2 days [17]. For the sake of simplicity, other medical or opportunity costs incurred due to adverse effects of immunization were not included in this study. Vaccine coverage was assumed to be 86.9% [9]. The discount rate for direct and indirect costs was 0%. The primary vaccine failure rate was 3.5%. For simplicity, the secondary vaccine failure rate was not counted.

Age distribution follows this data set.

To simplify analysis, the number of patients was set at 100,000 per year for the whole country. We adopted the lower limit of the estimated number of patients [3,5]. For other data including upper limit, we estimated by sensitive analysis.

The fatality rate for measles in Japan is 1/10,000. As for severe complications, encephalopathy/encephalitis occurs in 1/1,500 cases and subacute sclerosing panencephalitis occurs in 1/100,000 cases [18].

Our estimation is based on the assumption that the number of measles patients will be zero after a 13.1% increase (from 86.9% to 100%) in vaccine coverage. In other words, the vaccination cost is that which is incurred in increasing vaccine coverage by 13.1% to reach 100% coverage. The benefit is the reduction of the direct and indirect costs attributed to measles infection.

The BCR is described as follows:

BCR = [*Vaccine cost to increase 13.1% coverage*]/[*Reduction of direct and indirect costs of measles infection*].

### Parameter settings for sensitivity analysis

To conduct sensitivity analysis, alternative parameter values were arranged in the probable distribution.

The alternative values of each parameter were set as follows.

a) Total number of patients: 100,000, 150,000 and 200,000 per year.

b) Case fatality rate: 1/10,000, 5/10,000, 10/10,000.

c) The rate of admission among adult patients: 70, 80, and 90%. For pediatric patients: 30, 40, and 50%.

d) Vaccination cost: JP¥ 5,000, 6,000 and 7,000 (US$ 42.1, 50.5, and 58.9).

e) Discount rate: 0, 1 and 3%.

Because some parameters may change simultaneously, we conducted multidimensional sensitivity analysis based on the assumption that the probabilities of occurrence of all of the parameters are the same. Based on this assumption, we analyzed 243 combinations to estimate direct and indirect costs estimation and calculate BCR. For estimation of nationwide costs for measles treatment, we applied estimated direct costs and indirect costs to assumed number of patients following age distribution of measles patients.

### Ethical consideration

Before beginning the survey, the ethical committee of the Chiba-Nishi General Hospital, represented by the director of the hospital, formally approved the research. Although the nature of this study meant that some potentially sensitive information was required, we took great care in maintaining the privacy of the patients involved in our investigation. In each step of data collection and processing, personally identifiable information that was no longer required was deleted. This included patient name, telephone number, patient/parental employment status, and address.

## Results

### Identification of measles patients

The inpatient/outpatient ratio of extracted 291 candidates was 1.43:1.00 (171:120). A total of 194 cases matched our criteria; 97 cases were excluded because their records did not match our diagnostic criteria.

Three pediatricians, who were in charge of treatment to 34 families, conducted the telephone interviews. To confirm the parental employment status, the physicians conducted 14 inpatient family interviews and 10 outpatient family interviews; 1 inpatient family and 5 outpatient families refused. The remaining 10 interviews were conducted to clarify the clinical course of each patient and also included the question on parental employment status. At the time of the interview, the prognoses of all the patients were confirmed. While parent(s) did not remember the exact date of rash onset, they remembered that rash first appeared on the face and then spread to trunk. Most decided to discontinue treatment after spontaneous fever resolution.

In total, 194 measles patients were identified using the available data; 94 patients recovered without hospitalization; and 100 cases were admitted to hospital. Adult patients were more likely to be admitted to hospital than pediatric patients. Regarding diagnosis, 132 cases were laboratory confirmed (inpatient: outpatient = 78:54) and 62 cases were confirmed by Koplik spots (inpatient: outpatient = 22:40). For those who were diagnosed with measles only by Koplik spots, the clinical course was examined by the survey team to clinically confirm the diagnosis.

No patients were identified with severe brain damage (encephalitis) nor did we identify any measles-related deaths.

### Sex and age distribution of measles patients

**Figure 2:**
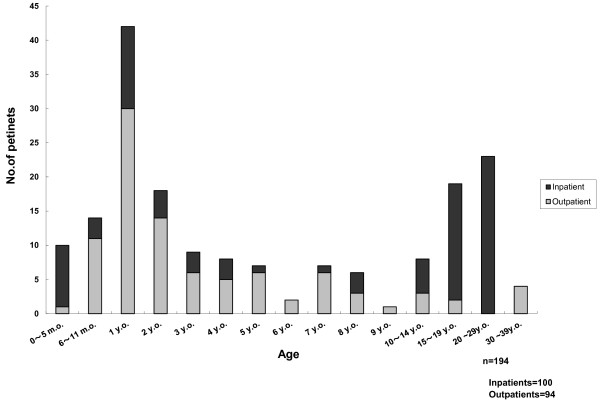
**Age distribution of whole patients**.

The male: female ratio was 1.49:1.00 (116:78). Two peaks were observed in age distribution (1 year and > 20 years: Figure 2). The highest peak was the 1-year-old cohort; the 20-29-year-old cohort was the 2^nd ^peak. Adult patients were more likely to be admitted to hospital than pediatric patients.

### Treatment and examination

Treatment for non-hospitalized cases included oral cough drugs (n = 85), beta stimulant drugs for bronchitis cases (n = 10), infusion for dehydration cases (n = 54), and inhalation of beta stimulants for dyspnea (n = 10). Tests performed were as follows: blood test, including blood cell count and general biochemical tests (n = 54); measles IgM antibody titer (n = 54); chest X-ray for diagnosis for ruling out of pneumonia (n = 10); brain computed tomography scans for complicated febrile convulsion cases (n = 2); and abdominal ultrasonography for severe diarrhea cases (n = 12).

Patients were usually hospitalized if they were suffering from severe dehydration, systemic malaise, or dyspnea. In all hospitalized cases, patients received drip infusion for correction of dehydration (n = 100) and systemic antibiotics against complicated bacterial infection (n = 100). In cases of severe pneumonia or those complicated by bronchial asthma, oxygen therapy was provided (n = 4) in addition to beta stimulant inhalation therapy (n = 45) and oral cough drugs (n = 70) and oral beta stimulants (n = 45). Systemic steroids were administered in cases of severe hypoxemia with interstitial pneumonia cases (n = 1). Tests performed for inpatients were as follows: blood test, including blood cell count and general biochemical tests (n = 78); measles IgM antibody titer (n = 78); chest X-ray to rule out pneumonia (n = 45); brain computed tomography scans for complicated febrile convulsion cases (n = 2); and abdominal ultrasonography for severe diarrhea cases (n = 26).

No cases involved administration of vitamin A or gamma globulin.

### Costs

**Figure 3:**
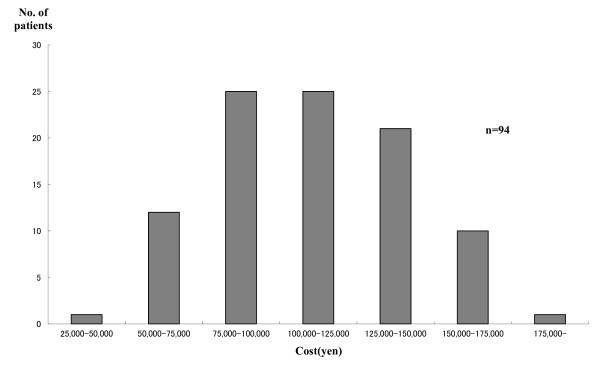
**Distribution of treatment cost of outpatients**.

**Figure 4:**
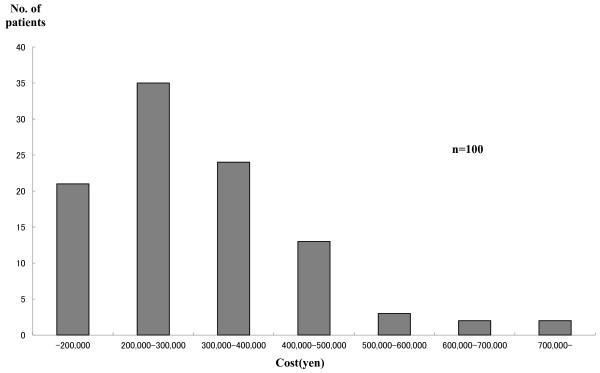
**Distribution of treatment cost of inpatients**.

The distribution of total costs is presented in Figure 3 (outpatients) and Figure 4 (inpatients). The average treatment cost of 94 outpatients was US$ 1010.1 (JP¥ 120,000). For the 99 inpatients it was US$ 2,525.30 (JP¥ 300,000). The percentage of indirect costs was much higher in outpatient cases. Based on estimated direct and indirect costs, the nationwide costs of measles treatment and universal vaccination were estimated. The percentage of indirect costs borne by outpatients was higher than that of inpatients.

Assuming that the number of measles patients is 100,000 per year nationwide, the average cost (including direct and indirect costs) for measles treatment was estimated as US$ 404 million (JP¥ 48.0 billion). Assuming that the number of children targeted for vaccination is 1,200,000, the estimated nationwide cost (including direct and indirect costs) for single-dose vaccination was estimated as US$ 165 million (JP¥ 19.6 billion) (Table 4).

**Table 4:** Distribution of costs for measles treatment and vaccination for whole Japan

		Whole cost (Direct cost + Indirect cost)	Direct cost	Indirect cost
Measles Treatment	Mean	404.7	394.4	10.4
				
	Minimum	76.3	76.3	0.0
	25%	86.2	85.8	0.4
	Median	174.0	171.6	2.4
	75%	198.3	191.2	7.2
	Maximum	2,550.4	2,515.5	34.9

Vaccination	Mean	165.1	163.1	1.9
				
	Minimum	154.8	153.4	1.4
	25%	154.8	153.4	1.4
	Median	155.6	153.4	2.3
	75%	193.4	191.1	2.3
	Maximum	210.9	209.4	1.5

The unit of the table is per million US$ (1 US$ = 118.8 yen)

Direct and indirect costs for measles treatment of vaccination were estimated by sensitivity analysis.

The result of sensitivity analysis showed negatively skewed distribution.

### Benefit cost ratio (BCR)

The estimation of BCR in the base case and sensitivity analysis is shown in Table 5. In the base case, BCR was 2.48. Sensitivity analysis showed a minimum of 2.21 and a maximum of 4.97. Multi-dimensional sensitivity analysis showed a median of 4.20, an average of 4.20 and a 95% confidence interval of 2.49-6.17.

**Table 5:** Sensitivity analysis of BCR (Benefit Cost Ratio)

Total No. of Patients (/10,000)	Case Fatality Rate (/10,000)	% of Admission (adults) (%)	% of Admission (children) (%)	Vaccine cost /1,000 yen	Discount Rate (%)	BCR
10	1	80	30	5	0	**2.48**
15	1	80	30	5	0	3.63
20	1	80	30	5	0	4.97
10	1	80	30	5	1	2.21
10	5	80	30	5	0	2.69
10	10	80	30	5	0	2.43
10	1	70	30	5	0	2.30
10	1	90	30	5	0	2.53
10	1	80	40	5	0	2.62
10	1	80	50	5	0	2.56
10	1	80	30	4	0	2.58
10	1	80	30	6	0	2.27
10	1	80	30	5	3	2.35

Note: The underlined BCR value **2.48 **indicates the BCR for base case.

## Discussion

### Age distribution

Our survey revealed two peaks in age distribution: 1 year and 20-29 years. In our data, 1 year-old-cohort was the highest of these two peaks. In the national surveillance data, the 10-14-year-old cohort was the 2^nd ^peak. The endemic patterns were almost the same as the endemic patterns in Japan in 1999-2003 [8].

### Estimated Costs

#### Costs as burden of disease

We estimated the total cost of measles treatment in Japan to be US$ 404 million (JP¥ 48.0 billion). This estimation contains both direct and indirect costs; however, US$ 404 million can be considered a serious economic impact on its own. The additional costs are incurred because measles is a wasting disease and measles patients require continuous treatment for dehydration, malaise or dyspnea even when a patient is not admitted to hospital.

#### Economic effectiveness

Our estimation of measles BCR was 2.48 in the base case and sensitivity analysis showed a minimum of 2.21 and a maximum of 4.97.

Benefit cost analyses of measles vaccination (or MMR) have been conducted in several countries. However, almost all of these analyses deal with a two-dose vaccine policy, which reflects the policies of each country. Among these analyses, the earliest measles studies that involve one-dose vaccination found the BCR in Austria to be 4.48 [19], and that in Finland to be 3.16-3.88 [20]. In a study in a hypothetical European country using single-antigen measles vaccine, the incremental benefit of the two-dose regimen compared with one-dose regimen varies from €1.2 million to €1.83 million [21]. In a Canadian study of single-antigen measles vaccines, the incremental net benefit of a two-dose regimen compared to a one-dose regimen is CN$ 0.18 billion and concluded that the two-dose regimen was favorable [22]. A study in U.S.A. showed that the BCR for direct costs and BCR for direct and indirect costs were 14.2 and 26.0, respectively. In this study, net savings are also calculated and were found to be US$ 3.5 billion in direct costs and US$ 7.6 billion if direct and indirect costs were combined. From the perspectives of BCR and net savings, the national 2-dose MMR vaccination program was concluded to be highly cost-beneficial [23].

It is noteworthy that at present cost effectiveness is used to find the best options for vaccination strategy. Simons *et al *estimated incremental costs and cost effectiveness of user-defined vaccination strategy using a measles strategic planning tool that was developed to facilitate analysis of national immunization and surveillance data and cost effectiveness of different vaccination strategies [24]. In a Ugandan study, Bishai *et al *compared supplementary immunization activities using dynamic stochastic model with other interventions including malaria and African trypanosomiasis control using the incremental cost effectiveness ratio as an indicator [25].

Although the small number of one-dose measles vaccine studies makes comparison difficult, other types of vaccine can be considered. In China, the BCR for universal hepatitis B vaccination was found to be 1.4 [26]. The BCR for vaccinating preschool children against influenza in United States was 1.93 [27]. Two adult studies in the same country found the BCR for vaccinating healthier adults against influenza to be 1.81 [28] and 2.92 [29].

### Validity of BCR

#### Assumptions of analysis

In our analysis, we did not include fees for patients to be placed in private hospital rooms. However, since measles patients are usually isolated to control nosocomial infection, private room fees should be considered in the estimation of social costs. If the room fee of US$ 67.3 (JP¥ 8,000 yen) in Chiba-Nishi hospital applied to this study, the BCR could increase by about 0.05-0.1. Although it is controversial whether private room fees should be included in the estimation, the affect of the fees would seem to be very small.

We assumed that 2 workdays would be lost to vaccination or nursing for mild side effects from vaccination. This assumption is based on the fact that 20% of vaccine recipients suffer from mild fever for approximately 2 days. If less than 2 workdays are lost, the indirect cost of vaccination would decrease and the BCR value would increase.

Furthermore, mild cases of measles should be considered. Such patients may be treated at home and recover without visiting hospital. It is difficult to extrapolate the number that these cases represent. However, even mild cases require family care and absence from work. With these considerations, the BCR could also rise, further highlighting the benefits of vaccination.

### Limitations

Our study has a number of limitations. A major limitation is our use of Matsudo City as a proxy to represent all of Japan and our use of Chiba-Nishi General Hospital as a representative proxy for Matsudo City. We believe that this is reasonable based on the age distribution of patients discussed earlier in this discussion. However we can speculate that less serious measles cases might be treated in general physicians' clinics, and that cases observed in Chiba-Nishi general hospital could be the more serious measles cases. Thus, the cases that we observed in this study might be more serious than the actual situation. To avoid these imitations, a multicenter study including general physicians should be considered as a next step and should be addressed in future studies. In reality, it is difficult to obtain data necessary for this kind of analysis because it inevitably requires individual information. A solution to this issue is something that should also be addressed in the future.

We assumed that all the immunized children would have fever for two days in spite of the fact that only 20% of vaccine recipients suffer from mild fever lasting for approximately 2 days. This may be an excessive assumption, but it does not change the conclusion of the study. If the percentage of vaccine recipients suffering from fever or the duration of fever were reduced in the model, then the BCR would increase because the indirect cost of vaccination would fall.

There is also a possibility that the telephone interviews were affected by recall bias. In the planning stage, our survey team discussed this concern. In fact, 10 interviewees could not remember the exact date of rash onset. Even though these results do not have a significantly negative impact on our survey, they should be noted.

In addition, we did not include direct costs of over the counter drugs, printing materials or human resource allocation for campaigns and so on which may be necessary to advertise the importance of measles vaccine. We also did not consider indirect costs of special education for subacute sclerosing encephalitis. If we were to include these elements, the indirect cost of vaccination would likely increase and the BCR would likely decrease. Despite the difficulties in estimating these kinds of cost, it is something that we aim to investigate in a more detailed estimation. Furthermore, we did not consider secondary vaccine failure. Including the possibility of secondary vaccine failure would provide for more precise analysis.

Finally, the present study involved purely static analysis. This study is based on the assumption that the number of measles patients would be zero if all 1-year-old cohorts received the vaccine. However, since it is well known that approximately 5% of people do not develop protective antibody levels after just one dose of measles vaccine, 100% measles vaccine coverage achievement would not establish herd immunity high enough to prevent sporadic measles transmission. In a practical sense, it is impossible for measles cases to be reduced to zero immediately after 100% coverage has been achieved. In this sense, this study analyzes stable situations like that of polio. In reality, when 1-year-old cohorts are vaccinated and herd immunity increases, the decrease in measles incidence is gradual. Over time, the costs caused by measles epidemics could be significantly reduced and eventually, even averted. This type of model, which includes an adjustment process, is called dynamic analysis and can produce a markedly different outcome from static analysis [14]. For that purpose, it is necessary to consider the influence of vaccine coverage on epidemics. For example, a study in the United States reported that regional measles epidemics raise vaccine coverage and influences the timing of vaccinations [30]. However, it is difficult to arrange datasets for dynamic analysis. Thus, we conducted static analysis using the best data that could be obtained.

This is a limitation of both our study and the NIID recommendation. In the future, the interrelationship between epidemics and vaccination coverage should be clarified in a dynamic framework. Our survey involves benefit cost analysis that counts labor loss and severe subsequent complications as the only indirect cost. While benefit cost analysis is relatively easy to calculate, we did not analyze the impact of other disutilities such as mental stress. Cost utility analysis is ideal and widely utilized for this purpose [31]. In order to apply such analysis to the present study, we would need far more precise data than were available.

## Conclusion

We estimated Japan's measles disease burden in the measles endemic era of 1999-2003 and evaluated the effectiveness of immunizing children soon after their 1-year birthday. The total measles treatment cost was found to be US$ 404 million with a BCR of 2.48 in the base case. With consideration to the impact of the measles disease burden, our study found the recommendation of immunizing children soon after their first birthday to be suitable and effective.

## Competing interests

The authors declare that they have no competing interests.

## Authors' contributions

KT was the principal investigator of the study. KT and JYK were responsible for retrieving the data and KT and YO were responsible for analyzing the data. KT drafted the article, and YO supervised the contents of article. All authors read and approved the final manuscript.

## Pre-publication history

The pre-publication history for this paper can be accessed here:

http://www.biomedcentral.com/1472-6963/11/254/prepub

## Supplementary Material

Additional file 1 **Technical Annex "Estimation of wage functions"**. A brief summary of the procedure of indirect cost estimation. See Table 2 &3 for reference. Click here for file
