# Supplementary material for: The economic disease burden of measles in Japan and a benefit cost analysis of vaccination, a retrospective study
Source: BMC Health Serv Res. 2011 Oct 7;11:254. doi: 10.1186/1472-6963-11-254 (PMC3217873; doi:10.1186/1472-6963-11-254)
Supplement: Additional file 1 — Technical Annex "Estimation of wage functions". A brief summary of the procedure of indirect cost estimation. See Table 2 &3 for reference. [file 1472-6963-11-254-S1.DOC]

**Technical Annex 　”Estimation of wage functions”**

*Estimation of wage functions*

In order to estimate indirect costs, we first needed to calculate the wage function. Wage was estimated by the traditional labor economics method, which is the weighted least square with the number of labors as weight. The wage function was determined for male/female full-time workers and female part-time workers, based on the wage of the age-group data from the Japanese wage census survey (1998 version). For full-time workers it is expressed by the logarithm of monthly scheduled cash salary (yen)/1,000 for full-time workers. For part-time workers it is the logarithm of scheduled cash salary (yen) per hour. Using the number of workers as weight, the weighted least-squares method was applied to the estimation.

The results are shown in Table 2. In Table 2, the number of samples is 12 because the wage census survey was divided age into 12 groups. The function of full-time workers is expressed as the quadratic function of age, thus its estimated value is applied for wage after the index transform. For part-time workers, neither age nor quadratic function was significant. It was therefore concluded that the index transform of the constant 6.78 is appropriate for the wage.

*Assumptions of indirect costs*

In order to estimate indirect costs for patients and attendants, we applied the human capital approach. For this estimation, some assumptions were made.

In principle, for patients younger than 18 years, the patient’s own indirect costs were not counted. If the patient or attendants had no regular job, the indirect costs were assumed to be the same as those of a part-time worker. For patients older than 18 years, indirect costs were applied to both patients and attendants based on the estimated wage functions.

For hospitalized patients, we assumed that they visited hospital as outpatients 3 days before admission. This is because our survey data showed that the median interval of outpatient hospital visits was 3 days. We also assumed that patients take 3 days of home rest before admission and 3 days after discharge.

*Workdays lost by patients or family members*

The employment status was complemented by telephone interview.

For estimation of workdays lost by patients or family, 99 hospitalized cases were adopted as the sample because the data on details of the patient and parent(s)’ age or employment status were complete. The result is shown on Table 3.

For outpatient cases, since some patients recovered without hospitalization, patient or parent employment status was not always clear even after telephone survey. For these cases, regression on square and cube terms of parent’s age was applied for indirect cost estimation of inpatients and the predicted value of the square and cube terms of outpatient parent’s age was used for the indirect cost.
